# Supplementary figures and images for: Novel genes associated with folic acid-mediated metabolism in mouse: A bioinformatics study
Source: PLoS One. 2020 Sep 11;15(9):e0238940. doi: 10.1371/journal.pone.0238940 (PMC7485790; doi:10.1371/journal.pone.0238940)

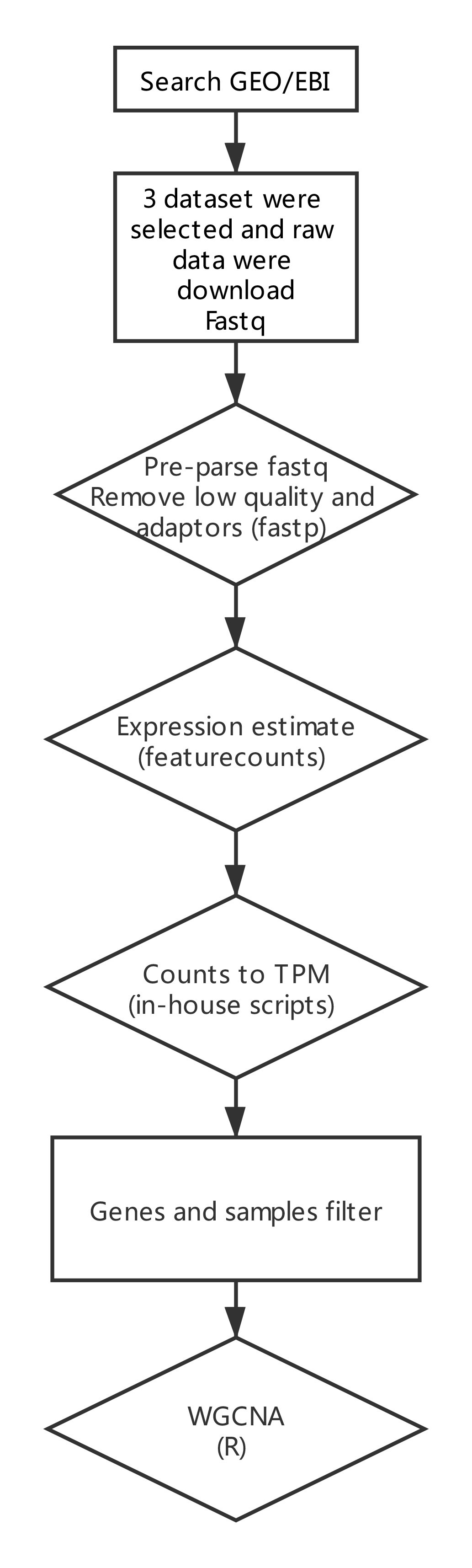

Supplement: S1 Fig — (TIF) [file pone.0238940.s002.tif]

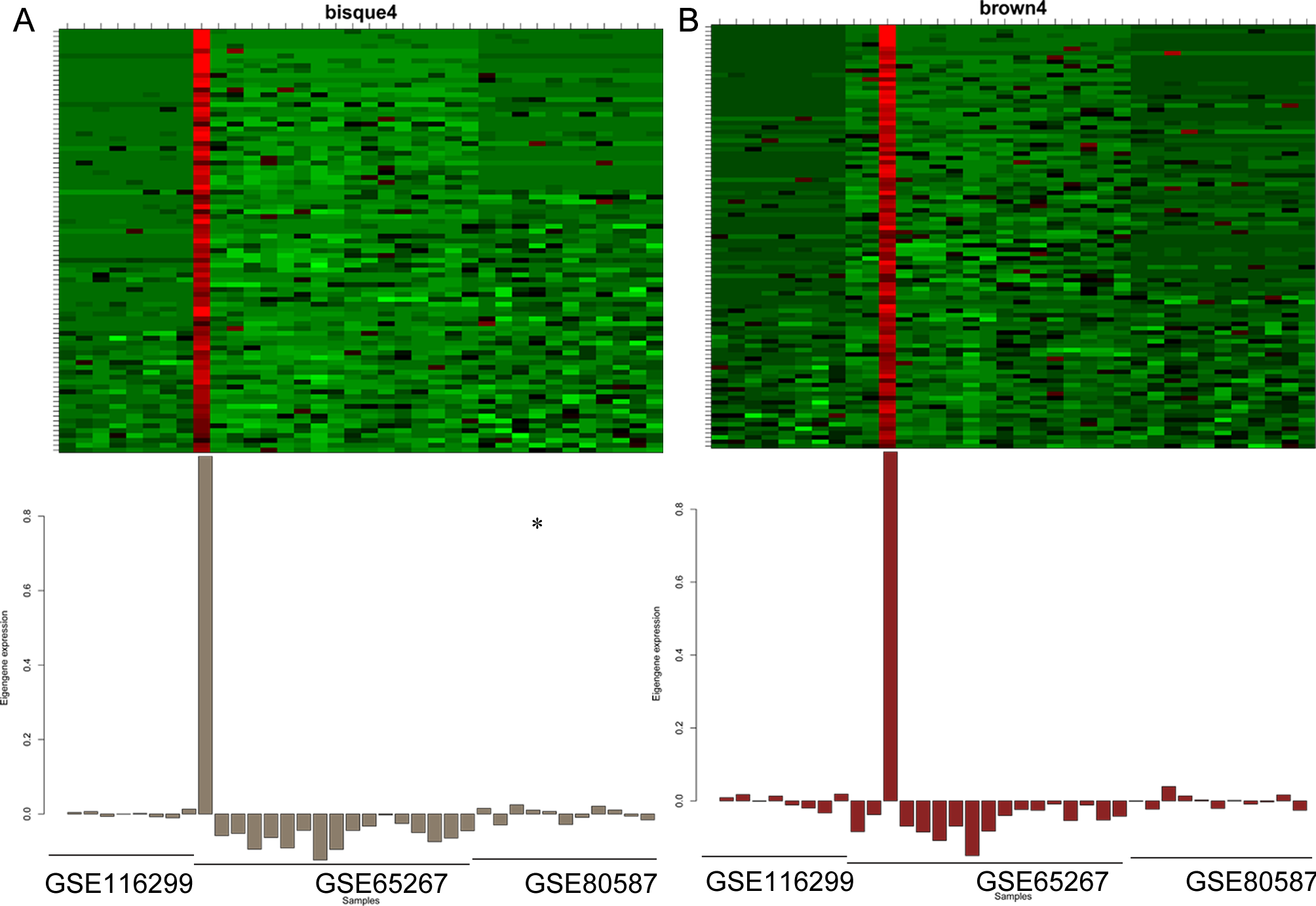

Supplement: S2 Fig — (TIF) [file pone.0238940.s003.tif]
