# Supplementary material for: Novel genes associated with folic acid-mediated metabolism in mouse: A bioinformatics study
Source: PLoS One. 2020 Sep 11;15(9):e0238940. doi: 10.1371/journal.pone.0238940 (PMC7485790; doi:10.1371/journal.pone.0238940)
Supplement: Attachment — Submitted filename: response to reviewers.docx [file pone.0238940.s005.docx]

Dear editor,

Thank you for giving us the opportunity to revise the manuscript. We gladly accept the reviewers’ comments and revise them one by one. We hope that this revised manuscript will meet the publishing requirements.

The point by point response were listed below.

Thanks for the editor's work, and the reviewers for their careful review.

Kind regards,

Tingxi Hu

Reviewer #1: The authors did a good job of addressing overarching concerns as well as specific prior issues raised by modifying the paper and directly describing the changes in response to the comments. Overall, I have no other significant concerns, but I did notice a few final typos to fix.

Final revisions:

• Line 34: “HubgenesHub genes” should be changed to just “Hubgenes” or “Hub genes”

• Line 84: “10and 14” should be fixed to “10 and 14”

• Line 162: “wasnoted” should be broken up into “was noted”

• 172: “97eigengenes” and “showedsignificant” should be split into “97 eigengenes” and “showed significant”

• 195: “Hubgeneswith” should be split up

• 223: “B12can” should be split up

**Response: Thank you for your comments. We have corrected all the typographical errors you mentioned and other errors in this article.**

• Figure 2D should be retitled “Number of nodes in modules” or something along those lines. Numbers is currently misspelled as numbers.

**Response:** **Thanks for your comment. We're sorry that the title is misspelled. The title is now adjusted to “Number of nodes in modules”.**

Reviewer #2: The authors addressed my comments. However, there are still some typographical errors.

These errors are all two words written together and they can be corrected easily by using a spellchecker.

pp1 line 34 HubgenesHub genes

pp6 line 195 hubgeneswith

pp6 line 200 weresimultaneously

pp6 line 202 wereidentified

pp7 line 241 promotestumor

pp8 line 273 mayhave

**Response:** **Thank you for your comments. We have corrected all the typographical errors you mentioned and other errors in this article.**
