# Supplementary material for: Novel genes associated with folic acid-mediated metabolism in mouse: A bioinformatics study
Source: PLoS One. 2020 Sep 11;15(9):e0238940. doi: 10.1371/journal.pone.0238940 (PMC7485790; doi:10.1371/journal.pone.0238940)
Supplement: Attachment — Submitted filename: Response to Reviewers.docx [file pone.0238940.s006.docx]

Dear Editor,

Thank you for your comments.

We have polished the manuscript as required. We have selected a third-party organization to polish the manuscript, and the polishing report has been uploaded.

Best regards

Hu
